# Effect of Pyrolysis Temperature on the Carbon Sequestration Capacity of Spent Mushroom Substrate Biochar in the Presence of Mineral Iron

**DOI:** 10.3390/molecules29235712

**Published:** 2024-12-03

**Authors:** Bin Liu, Zebing Xing, Yuxin Xue, Ji Zhang, Junlin Zhai

**Affiliations:** College of Agricultural Engineering, Shanxi Agricultural University, Jingzhong 030801, China; 20233058@stu.sxau.edu.cn (B.L.); 18235488991@163.com (Y.X.); zhangji9171018@163.com (J.Z.); 15563505149@163.com (J.Z.)

**Keywords:** pyrolysis temperature, mineral Fe, carbon retention, carbon stability, carbon sequestration

## Abstract

The preparation of biochar typically involves the pyrolysis of waste organic biomass. Iron-rich magnetic biochar not only inherits the characteristics of high specific surface area and porous structure from biochar but also possesses significant advantages in easy separation and recovery, which has shown great application potential in various fields such as soil improvement and water resource remediation. This study aims to explore the influence of mineral iron on the carbon sequestration capability of biochar during the pyrolysis process. Experiments were conducted by using spent mushroom substrates as raw materials to prepare biochar at different temperature intervals (300 to 600 °C). The addition of exogenous iron has been found to significantly enhance the carbon retention rate (12.2–44.5%) of biochar across various pyrolysis temperatures and, notably, improves the carbon stability of biochar at 300 °C, 400 °C, and 600 °C. Through the analysis of thermogravimetric mass spectrometry (TG-MS) and X-ray photoelectron spectroscopy (XPS), we discovered that iron catalyzes the thermochemical reactions and inhibits the release of organic small molecules (C_2_-C_5_) through both physical blocking (Fe_x_O_x_) and chemical bonding (C=O and O-C=O). The results of Raman spectroscopy and infrared spectroscopy analyses indicate that the addition of iron significantly promotes the graphitization process of carbon and enhances the thermal stability of biochar within the temperature range of 300 to 500 °C. When exploring the retention and stability of carbon during pyrolysis, it was found that under the conditions of 600 °C and the presence of iron, the maximum carbon sequestration rate of biochar can reach 60.6%. Overall, this study highlights the critical role of iron and pyrolysis temperature in enhancing the carbon sequestration capacity of biochar.

## 1. Introduction

China, as the world’s largest producer and consumer of edible mushroom, accounts for over 90% of the global annual production, making the edible mushroom industry an indispensable part of China’s agricultural production [1]. The development trend of China’s edible mushroom industry is positive; according to the latest statistics, the total production of edible mushroom of China in 2022 exceeded 42 million tons [2]. However, there are certain issues in the cultivation process; namely, the cultivation materials are not fully utilized, resulting in a large amount of by-products. Improper management of these by-products could lead to resource waste and environmental pollution. Currently, the utilization efficiency of mushroom substrates in China is relatively low. Many mushroom growers resort to burning, discarding, or crushing the substrates after harvesting [3]. However, these methods promote the proliferation of bacteria, molds, and pests, exacerbating environmental pollution and posing potential safety threats to humans and livestock [4]. Therefore, it has become an urgent task to develop an efficient and environmentally friendly approach to achieve sustainable utilization of mushroom substrates.

Mushroom residues, as a widely existing secondary biomass waste, can be converted into biochar through pyrolysis in an oxygen-deficient atmosphere. The application of biochar to soil not only brings additional agricultural benefits but also enhances environmental value, specifically in improving the physical and chemical properties of soil and effectively remediating soil pollution [5,6]. It is noteworthy that the key properties of biochar, including its carbon content, chemical composition, and stability, are jointly determined by the type of raw material and the specific conditions of the pyrolysis process, which, in turn, determine its environmental functions [7]. Different raw materials and pyrolysis procedures yield biochar with heterogeneous elemental compositions, functional groups, and thermal stability differences, thereby highlighting the decisive impact of material selection and pyrolysis conditions on the carbon stability of biochar [8]. In the preparation of biochar, the pyrolysis temperature is considered as the most pivotal factor that significantly influences the pyrolysis process and the characteristics and properties of the resulting biochar [9]. For instance, biochar extracted from biomass pyrolysis at lower temperatures (300–500 °C) contains more oxygen-containing functional groups, while higher temperatures (500–700 °C) result in fewer functional groups but increased mineral concentrations and micropores [10]. Higher temperatures (600 °C) also lead to aromatization and the formation of graphite-like carbon structures [11]. However, studies on the carbon sequestration capacity of biochar have shown that its carbon retention rate is not satisfactory. For example, treating spent mushroom substrates at a moderate pyrolysis temperature of around 500 °C results in an average carbon retention rate of only about 30% [12,13].

The mushroom substate contains abundant mineral components, primarily including alkali metals (Ca, Mg, K, Na) and transition metals (Zn, Fe) [14,15], which have a non-negligible impact on the conversion of C during the pyrolysis process. Inorganic minerals catalyze the thermal decomposition of biomass, thereby affecting the yield of biochar, the distribution of products, and the carbon structure. A large number of inorganic elements (P, Si, K, Na, Ca, Mg, Fe) have been shown to enhance the carbon retention rate of biochar to varying degrees [7,16,17,18,19,20]. Biochar containing ferromagnetic iron is widely noted for its reusable properties and ease of recovery, often used in wastewater treatment, and also for recovering nutrients from biogas slurry, which can then be applied as fertilizer to improve soil properties [21,22]. Previous studies have shown that the addition of FeCl_3_ can increase the carbon sequestration rate of sludge-based biochar [23]. Moreover, the addition of Fe can enhance the antioxidant capacity and stability of biochar by forming organic metal complexes such as Fe-O-C on the surface of biochar [24]. However, most research has focused on the yield and composition of the products. The impact of mineral Fe on the evolution of microstructure and carbon sequestration in biochar from spent mushroom substate remains under-explored. Therefore, strategies should be developed to regulate the pyrolysis process to achieve the overall carbon sequestration capacity of biochar, while considering both carbon retention and carbon stability.

This study employed *Pleurotus ostreatus* substrate as a precursor to prepare iron-containing biochar using a simple impregnation-pyrolysis method. The effect of mineral Fe doping on the carbon retention rate and stability of the biochar post-pyrolysis was evaluated. Techniques such as thermogravimetric mass spectrometry (TG-MS), X-ray diffraction (XRD), X-ray photoelectron spectroscopy (XPS), Raman spectroscopy, and Fourier transform infrared spectroscopy were utilized to finely probe the microstructure of carbon, exploring the effects of Fe and pyrolysis temperature on carbon changes during the pyrolysis process. Ultimately, the optimal pyrolysis temperature for the conversion of mushroom substrate into biochar was determined, aiming to fill the gap in the study of the carbon sequestration capacity of mushroom biochar during the preparation of magnetic biochar with the addition of mineral Fe.

## 2. Results and Discussion

### 2.1. Basic Properties of the Biochars

Table 1 presents a comprehensive analysis of the biochar yield, ash content, and basic composition under different pyrolysis temperatures. Specifically, when the pyrolysis temperature was set at 300 °C, 400 °C, 500 °C, and 600 °C, the corresponding yields were 48.1%, 31.4%, 27.6%, and 25.2%, respectively. It can be observed that the yield of biochar showed a gradual decline with the increase in pyrolysis temperature. Furthermore, the study found that the addition of Fe increased the biochar yield by 18.5%, 45.9%, 50.0%, and 54.0% at pyrolysis of 300 °C, 400 °C, 500 °C, and 600 °C, respectively, indicating that the effect of Fe on enhancing biochar yield becomes more significant with increasing temperature. Moreover, the ash content of biochar increased by two–three times when Fe was added, indicating successful incorporation of mineral Fe into the biochar [25]. The C content of biochar generally ranges between 61.24% and 80.73%. In contrast, biochar treated with iron addition exhibits a lower C content, ranging from 58.14% to 75.82%. This reduction in carbon content is primarily attributed to the addition of Fe minerals, which increases the total mass of the biochar, thereby affecting the proportion of C content [26].

The stability of biochar is determined through the evaluation of its chemical properties, with the hydrogen-to-carbon (H/C) and oxygen-to-carbon (O/C) atomic ratios being crucial indicators of its aromaticity and polarity. The molar ratio of O/C, as a core parameter for assessing biochar stability, primarily reflects the high reactivity of biochar and the abundance of its oxygen-containing functional groups. Based on the recommendation by Spokas in 2010 [27], the O/C molar ratio can serve as a key indicator of biochar stability in soil; this study determined that when the O/C molar ratio of biochar is below 0.2, the half-life of the biochar is expected to exceed 1000 years; when the O/C molar ratio is between 0.2 and 0.6, the half-life of the biochar is approximately 100–1000 years; and if the O/C molar ratio exceeds 0.6, the half-life of the biochar is anticipated to be less than 100 years. Subsequently, Budai et al. [28] proposed that the H/C molar ratio could also be used as an indicator for assessing biochar stability. The H/C molar ratio can be further calculated by determining the degree of unsaturation or the number of C=C double bonds to estimate the number of aromatic rings. According to the European Biochar Certificate (EBC) standards, the O/C ratio and H/C ratio can reflect the stability of biochar, with the upper limit for the O/C ratio set at 0.4 and for the H/C ratio set at 0.7 [29]. The data presented in Table 1 reveal that the addition of Fe significantly reduced the O/C ratio of biochar under all temperature conditions and also decreased the H/C ratio at 300 °C and 600 °C. These results indicate that the addition of Fe aids in diminishing the aromaticity and polarity of biochar, thereby enhancing its stability [20].

### 2.2. Effect of Pyrolysis Temperature and Fe Addition on Carbon Retention

#### 2.2.1. Effect of Pyrolysis Temperature on Carbon Retention

**Figure 1:**
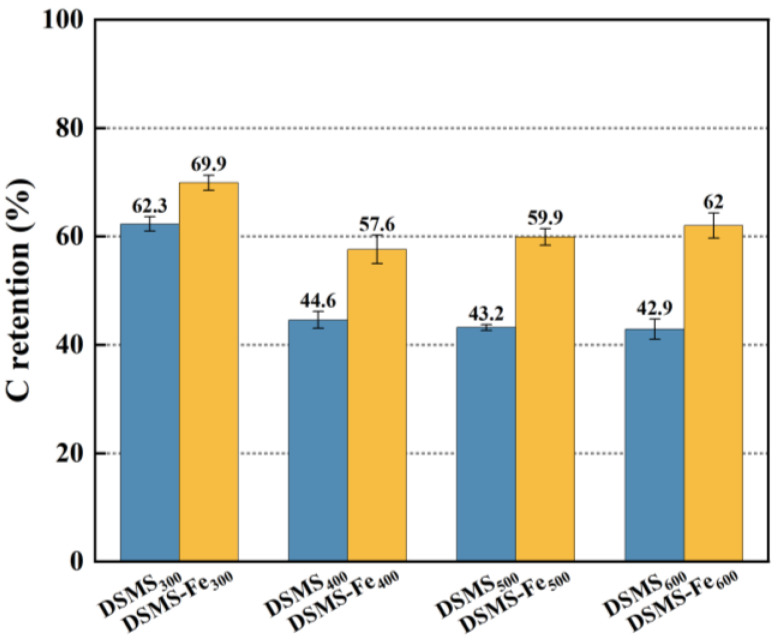
Carbon retention of DSMS and DMSM-Fe under different pyrolysis temperatures.

**Figure 2:**
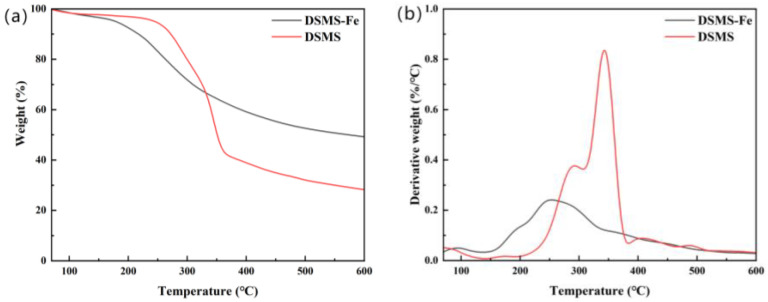
TGA (**a**) and DTG (**b**) curves of DSMS and DSMS-Fe combustion.

**Figure 3:**
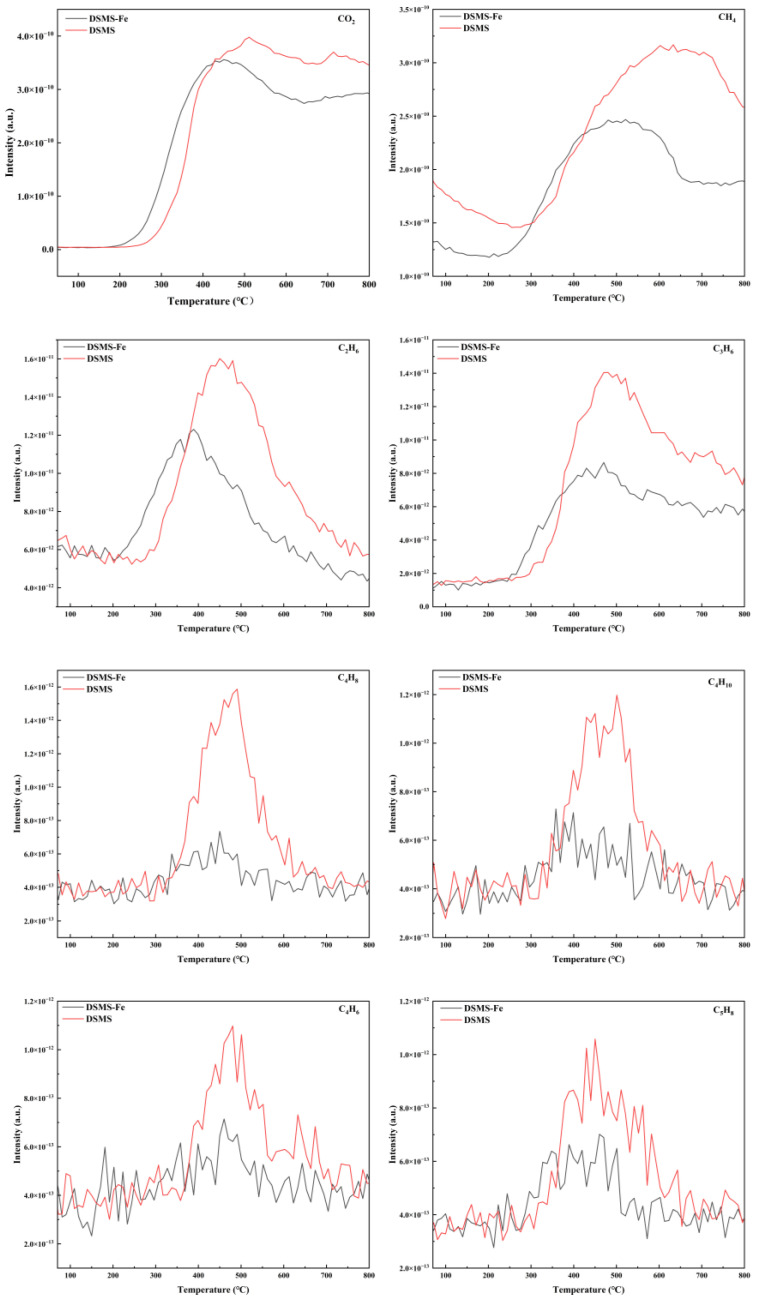
Release of small organic molecules during the DSMS and DSMS-Fe combustion.

The carbon retention performance of biochar is a crucial indicator for assessing its carbon sequestration capacity, which can be calculated according to Equation (2). As illustrated in Figure 1, the carbon retention rate of the raw biochar decreases from 62.3% to 42.9% as the pyrolysis temperature increases from 300 °C to 600 °C. This decline is primarily attributed to the release of additional carbon-based organic constituents during the pyrolysis [30]. The results revealed that the raw material exhibited mass loss phenomena at four specific temperature intervals, corresponding to volatile organic carbon (30–200 °C), unstable organic carbon (200–300 °C, primarily cellulose), refractory organic carbon (300–380 °C, primarily lignin and refractory carbon), and condensed refractory organic carbon (380–600 °C, lipids and aromatics). This finding is highly consistent with previous research results [31]. From Figure 2b, we can clearly observe two distinct peaks, which represent the degradation processes of (hemi)cellulose and lignin, respectively. The degradation peaks of (hemi)cellulose and lignin appear at approximately 300 °C and 350 °C, respectively. However, when the pyrolysis temperature exceeds 400 °C, we only observe minimal mass loss (Figure 2a). Therefore, a sharp decline in carbon retention rate is observed when the temperature is between 300 °C and 400 °C; in the temperature range of 400 °C to 600 °C, the decline in carbon retention rate is no longer significant (Figure 1). Additionally, the dynamic release behavior of small carbon-containing gases generated during pyrolysis was monitored using mass spectrometry. Figure 3 shows that these carbon-containing molecules are primarily released within the temperature interval of 300–600 °C, a result that aligns with the trend of reduced carbon retention in biochar.

#### 2.2.2. Effect of Fe Addition on Carbon Retention

Under all pyrolysis temperature conditions, the addition of mineral Fe significantly promotes the retention of carbon in biochar (Figure 1). The carbon retention rate increased from 42.9% to 62.3% in DSMS to 57.6% to 69.9% in DSMS-Fe. This phenomenon is consistent with previous research findings, which reported that by adding FeCl_3_ to chicken manure within the temperature range of 350–550 °C, the carbon retention rate in biochar could be enhanced from 33.9–57.8% to 52.5–62.6% [32]. Additionally, as the pyrolysis temperature increased, a significant enhancement in the retention of carbon in biochar due to Fe was observed. Specifically, when the pyrolysis temperature increased from 300 °C to 600 °C, the incorporation of Fe resulted in carbon retention rates that were 12.2%, 29.1%, 38.7%, and 44.5% higher than those without Fe addition, respectively. This indicated that the effect of Fe on carbon retention was associated with pyrolysis temperature. This result indicates a significant correlation between the effect of iron on carbon retention capability and the pyrolysis temperature.

Figure 2 illustrates the pyrolysis behavior of key carbon-based substances in DSMS-Fe. Notably, the addition of Fe significantly influences the thermochemical reaction peaks within the range of 100 °C to 400 °C. Specifically, the introduction of iron promotes the occurrence of drastic mass reduction phenomena earlier in the pyrolysis process [33,34], causing the onset temperature of significant mass reduction to drop from 200 °C to 150 °C. Additionally, the decomposition peak of (hemi)cellulose shifts from around 300 °C to approximately 200 °C, and the decomposition peak of lignin moves from around 350 °C to about 250 °C. Correspondingly, the peak heights of these decomposition peaks decrease relatively (Figure 2). These results reveal that the addition of Fe not only lowers the temperature threshold for carbon-containing substances decomposition but also slows down the rate of the decomposition process.

**Figure 4:**
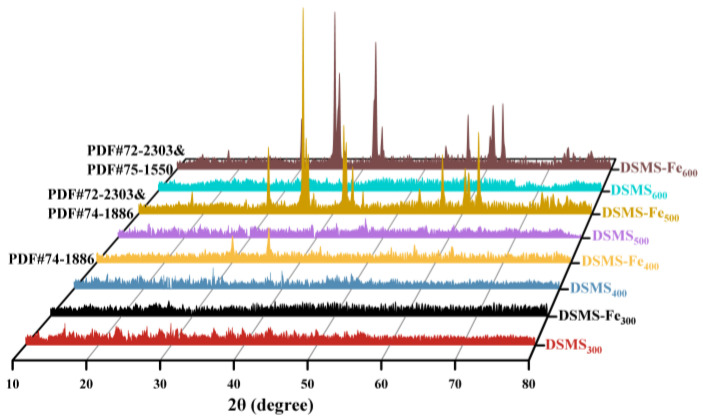
XRD patterns of DSMS- and DSMS-Fe-derived biochars produced at different pyrolysis temperatures.

Simultaneously, the dynamic release analysis of gases during pyrolysis reveals the significant impact of Fe addition on reducing the release of organic small molecules. It was observed that in the presence of Fe, the amount of carbon-containing small molecules (C_2_-C_5_) during the biochar release process was significantly reduced (Figure 3). This finding provides further compelling evidence for the mechanism by which Fe enhances the efficiency of carbon retention. In the XRD patterns (Figure 4), it can be observed that the DSMS does not exhibit any significant peaks. However, the DSMS-Fe shows a rich presence of Fe-containing crystals, including FeO and Fe_3_O_4_. Notably, these crystals only appear in the biochar within the temperature range of 400–600 °C, with no significant Fe peaks observed at 300 °C. At 400 °C, Fe predominantly exists in the form of FeO crystals, while at temperatures between 500–600 °C, Fe_3_O_4_ crystal peaks are prominent, indicating that Fe is transforming into magnetic Fe_3_O_4_ as the temperature increases. Concurrently, mineral Fe might function similarly to Ca, forming a uniform physical coating on the material surface and bonding with organic groups via chemical bonds (Fe-O-C) to form complexes [18,24], thereby enhancing the carbon retention rate in the biochar.

The above results reveal that the increase in temperature leads to a more significant role of Fe in promoting carbon sequestrations. Within the lower temperature range (200–300 °C), the presence of Fe primarily acts as a catalyst, accelerating the decomposition reactions, but its potential to enhance carbon retention efficiency has not been fully realized. However, within the higher temperature range (300–600 °C), the incorporation of Fe significantly enhances the carbon retention capacity of biochar.

### 2.3. Effect of Pyrolysis Temperature and Fe Addition on Carbon Stability

#### 2.3.1. Effect of Pyrolysis Temperature on Carbon Stability

**Figure 5:**
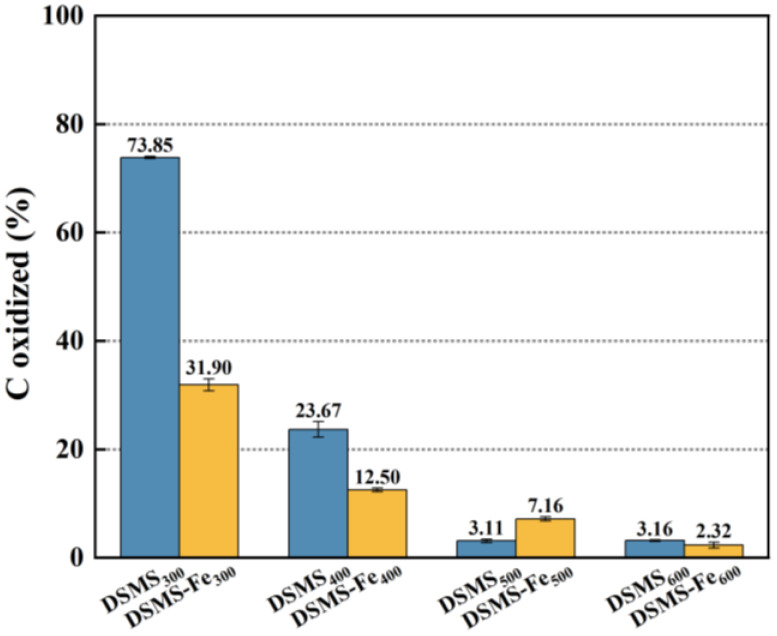
Carbon stability of DSMS and DMSM-Fe under different pyrolysis temperatures.

The stability of carbon can be considered as another crucial indicator for assessing the carbon sequestration capacity of biochar, which is typically evaluated through chemical oxidation methods. In this study, we employed the potassium permanganate oxidation colorimetric method with a concentration of 333 mmol/L to determine the content of easily oxidizable carbon in biochar. Figure 5 provides a detailed illustration of the carbon oxidized by KMnO_4_, reflecting the presence of unstable carbon in biochar. The stability of carbon in biochar is primarily determined by its carbon structure, which is closely related to the pyrolysis temperature: as the pyrolysis temperature increases, the stability of the carbon structure also enhances. Taking DSMS as an example, when the pyrolysis temperature increases from 300 °C to 600 °C, the content of unstable carbon decreases from 78.35% to 3.16% (Figure 5). Additionally, as verified by the data in Table 1, both the O/C ratio and H/C ratio exhibit a decreasing trend with increasing pyrolysis temperature. This phenomenon shows a significant negative correlation with the stability of biochar [35], thereby confirming the enhancement of biochar carbon stability.

**Figure 6:**
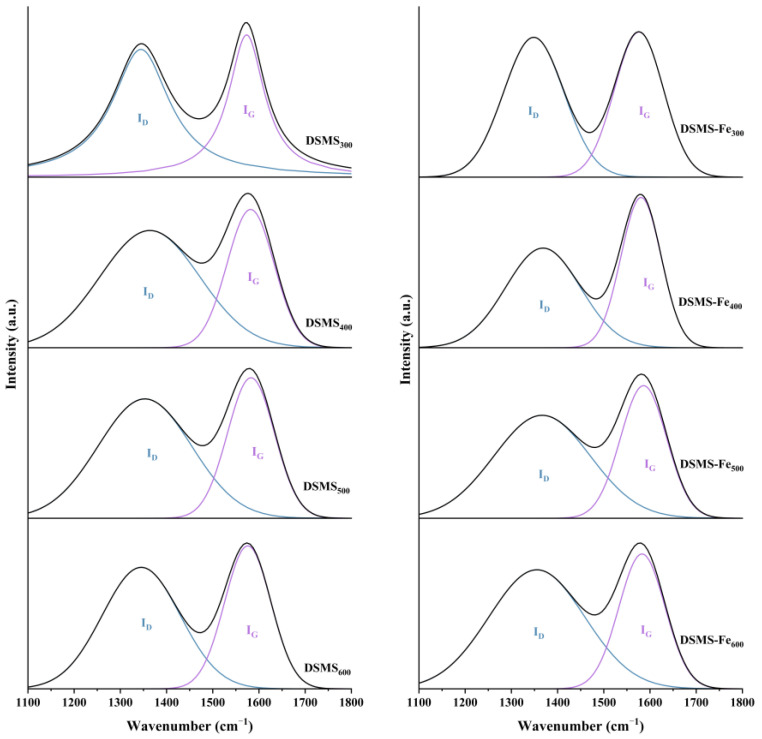
Raman analysis of DSMS and DSMS-Fe produced at different pyrolysis temperatures.

Raman spectroscopy analysis (Figure 6) reveals that the typical structure of sp^2^ carbon include two prominent peaks, the D-band and the G-band, which, respectively, represent amorphous and ordered carbon structures [36]. The value of the typical indicator I(D)/I(G) is negatively correlated with the graphitization degree and orderliness of the carbon structure; a lower value of I(D)/I(G) indicates that the biochar has a more complete and ordered graphite structure [37], thereby making the carbon structure more stable. As shown in Table 2, as the pyrolysis temperature increased from 300 °C to 600 °C, the I(D)/I(G) ratio of DSMS biochar exhibited a trend of first increasing and then decreasing. In fact, the increase in pyrolysis temperature is conducive to the transformation of amorphous carbon structures into graphitized carbon structures in biochar [38], thereby enhancing the stability of the biochar. Based on this, we infer that at pyrolysis temperatures exceeding 600 °C, the I(D)/I(G) ratio is expected to further decrease.

**Figure 7:**
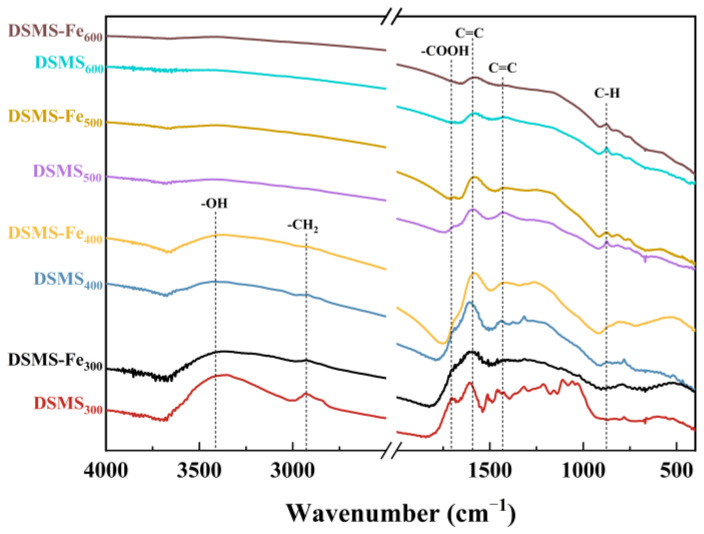
FTIR spectra of DSMS- and DSMS-Fe-derived biochars produced at different pyrolysis temperatures with major functional groups assigned.

The stability of organic functional groups on the surface of biochar is closely related to the stability of carbon, with their formation and evolution primarily dependent on the pyrolysis temperature. This study employed FTIR spectroscopy to investigate the transition processes of the main functional groups in depth (Figure 7). For DSMS, as the pyrolysis temperature increased, the stretching vibration of -OH gradually disappeared, indicating the evaporation of moisture and the cracking of organic functional groups [39]. When the pyrolysis temperature reached 500 °C, the peak at 2933 cm^−1^ disappeared, signifying the destruction of aliphatic -CH_2_, likely due to the decomposition of organic aliphatic hydrocarbons in the mushroom substate [40]. The band at 1702 cm^−1^, attributed to -COOH, was only present in biochar produced at a pyrolysis of 300 °C. The results revealed that, with increasing pyrolysis temperature, organic aliphatic hydrocarbon molecules decomposed, producing methane (CH_4_), carbon dioxide (CO_2_), and other small organic molecular gases. The disappearing -COOH might be converted into CO_2_. The MS curve for CO_2_ (Figure 3) confirmed this speculation, showing that CO_2_ was released when the pyrolysis temperature exceeded 300 °C. As the pyrolysis temperature increased, the bonds at 1590 cm^−1^ and 1430 cm^−1^ (aromatic C=C stretching vibrations) weakened, while the bond at 875 cm^−1^ (aromatic CH out-of-plane vibration) strengthened.

**Figure 8:**
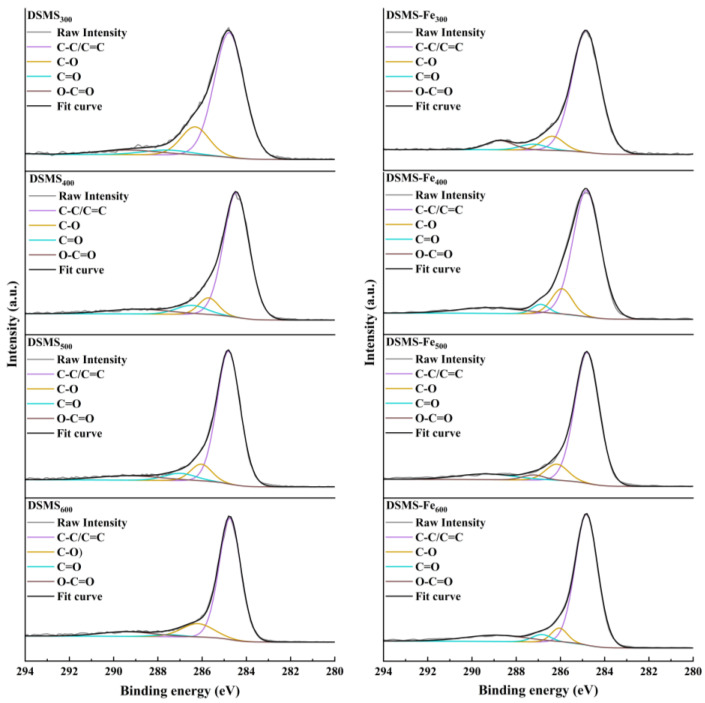
XPS analysis of the biochars produced at different pyrolysis temperatures.

To gain a deeper understanding of the carbon structural characteristics in biochar, this study utilized XPS spectroscopy for semi-quantitative analysis of the composition and content of carbon clusters (Figure 8). The C1s spectra of biochar with and without Fe addition could be deconvoluted into four peaks: C-C/C-C (284.3~284.7 eV) [41,42,43], C-O (286.2~286.8 eV) [44,45], C=O (287.2~287.5 eV) [46,47], and O-C=O (288.8~289.4 eV) [45,47,48]. The corresponding relative percentages of peak areas were listed in Table 3. The results indicated that as the pyrolysis temperature increased from 300 °C to 600 °C, the composition of carbon in biochar primarily shifted towards C-C/C=C groups, with a significant increase in their content. Concurrently, the proportion of carbon groups containing oxygen impurities, such as C-O, decreased markedly, while the proportions of C=O and O-C=O groups increased significantly. This phenomenon suggests that higher pyrolysis temperatures promote the removal of impurity atoms, allowing more carbon atoms to aggregate into pure carbon structural frameworks [49]. These pure carbon structural frameworks exhibit higher stability and antioxidation properties, further validating the enhanced stability of biochar at higher temperatures.

#### 2.3.2. Effect of Fe Addition on Carbon Stability

During the pyrolysis of mushroom substates, the addition of Fe significantly enhanced the carbon retention in biochar (Figure 1) and increased its relative carbon stability. Specifically, the unstable carbon content in DSMS-Fe_300_, DSMS-Fe_400_, and DSMS-Fe_600_ decreased to 31.9%, 12.5%, and 2.315%, respectively (Figure 5). Based on the results obtained at various temperatures in this study, and previous research indicating that mineral modification of biochar enhanced the stability of soil aggregates and soil carbon sequestration capacity [50]; exogenous minerals Fe was conducive to the formation of ordered carbon structures, thereby enhancing the carbon stability of biochar. Raman spectroscopy analysis indicates that the addition of Fe significantly reduces the I(D)/I(G) ratio of the resulting biochar under conditions of 300 °C, 400 °C, and 500 °C [51]. This result indicates that the presence of Fe promotes the formation of graphitic carbon and reduces the generation of defects [52].

Through the analysis of XPS, we found that the addition of FeCl_3_ into DSMS to resulted in the relative contents of C-C and C=C in the biochar being 82.44%, 76.65%, 79.74%, and 79.47% during the pyrolysis processes at 300 °C, 400 °C, 500 °C, and 600 °C, respectively. These showed relatively minor variations compared to DSMS. However, for DSMS-Fe, the relative percentage of C-O showed a decreasing trend, while the contents of C=O and O-C=O groups exhibited an increasing trend. This indicates that C-O is being converted to C=O and O-C=O. Therefore, the primary transformation pathways for carbon-containing groups are as follows: C-O → C=O and O-C=O → C-C/C=C. The mineral Fe may catalyze the oxidation of single-bonded C-O functional groups, leading to the formation of more C=O and O-C=O. These groups not only act as physical barriers but also interfered with the carbonation of these C-O and O-C=O groups, further reducing the content of C-C/C=C [53]. These effects were more pronounced at higher temperatures (Table 3). Thus, although Fe promotes higher levels of carbon retention during the biochar preparation process, especially at higher temperatures, more C=O and O-C=O could be detected from biochar preparation at higher temperatures. However, the pyrolysis temperature used may not yet have reached the level required to further convert these functional groups into more stable C-C/C=C structures.

### 2.4. Pyrolysis Temperature for Maximum Carbon Sequestration

**Figure 9:**
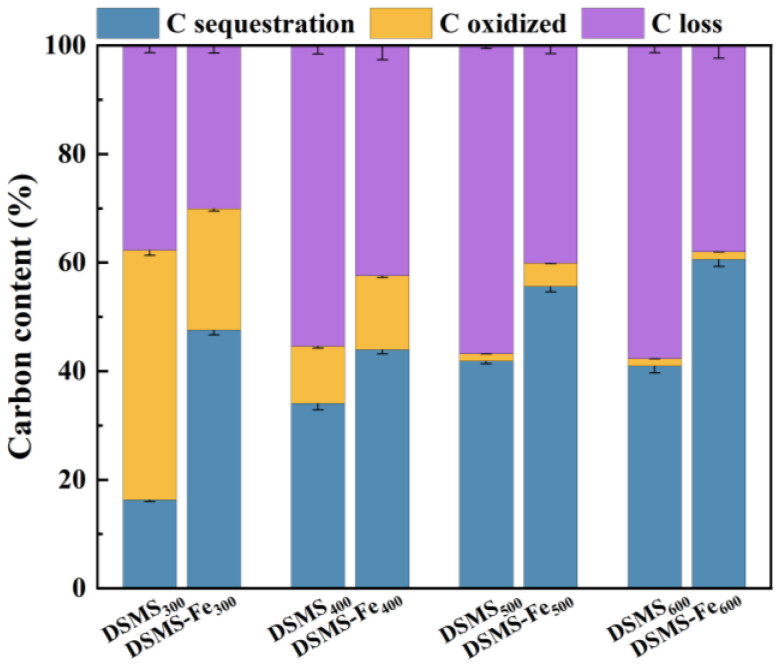
Contents of lost C, oxidized C, and sequestrated C in the biochars produced at different pyrolysis temperatures.

The evaluation of biomass carbon sequestration capacity should comprehensively consider both the carbon retention ability during the biomass pyrolysis process and the carbon stability of the biochar. Undoubtedly, in the absence of Fe doping, the carbon retention rate of biochar exhibits a decreasing trend with increasing pyrolysis temperature, while simultaneously enhancing its carbon stability to varying degrees. To accurately assess the effect of pyrolysis temperature and mineral Fe on carbon sequestration during the pyrolysis process, we normalized the initial carbon content of DSMS and DSMS-Fe before pyrolysis [53]. In Figure 9, the initial carbon content in the raw material is set at 100%, and the remaining carbon percentage after subtracting the carbon lost during pyrolysis and the carbon easily oxidized in the oxidation test is used to measure the carbon sequestration capacity in biochar during the pyrolysis process. The results indicate that as the pyrolysis temperature increases from 300 °C to 600 °C, the carbon sequestration in biochar increases from 16.3% to 41.9%. The term “final carbon sequestration” here takes into account both “carbon retention” and “carbon stability.” With increasing temperature, the increment in biochar carbon sequestration gradually diminished; the largest increase occurred within the 300–500 °C temperature range, while it even decreased within the 500–600 °C range. The presence of mineral Fe enhanced the biochar’s carbon sequestration rate from 40.9% to 60.6%, and this rate continued to increase as the pyrolysis temperature rises from 300 °C to 600 °C.

The carbon sequestration enhancement effect of Fe is most significant at 300 °C and 600 °C. Figure 9 indicates that for biochar prepared at 300 °C, 400 °C, 500 °C, and 600 °C, Fe, respectively, increased the final carbon sequestration by 31.3%, 13%, 13.7%, and 19.6%. Finally, it is clear from Figure 9 that the maximum carbon sequestration (60.6%) was achieved at 600 °C with the addition of Fe. This reveals a significant phenomenon that has long been overlooked by researchers: the introduction of exogenous Fe not only imparts magnetic properties to biochar but also significantly enhances its carbon sequestration capacity, albeit with notable differences in enhancement across various temperatures.

## 3. Environmental Application

Carbon sequestration, as an emerging technology for greenhouse gas reduction, directly constrains the emission of carbon dioxide into the atmosphere, thereby contributing to the reduction of atmospheric greenhouse gas concentrations. The application of biochar is a key component of this process, influencing greenhouse gas emissions through the stages of feedstock supply, pyrolysis, and application, demonstrating significant potential for atmospheric carbon sequestration on a global scale [54]. Therefore, enhancing the carbon retention and stability of biochar during the pyrolysis process is crucial for significantly reducing greenhouse gas emissions. According to statistics, the maximum emission reduction potential of biochar is estimated to be up to 6.6 Gt CO_2_ yr^−1^, which could offset up to 15.9% (41.6 Gt CO_2_ yr^−1^) of the carbon dioxide emissions generated by human activities [55,56]. This study proposes a straightforward approach by incorporating iron into biomass to enhance its carbon retention and stability, as well as to impart reusability. This method provides significant guidance for the production and application of biochar. In addition to improving carbon sequestration, biochar enriched with iron also demonstrates unique application values in other fields, offering multifaceted environmental benefits. Iron-based biochar composites can effectively remediate chromium-contaminated soils, significantly reducing the toxicity and mobility of chromium in the soil [57], and can adsorb cadmium and arsenic in rhizosphere soil and pore water, thereby decreasing the uptake of these elements by plant roots [58,59]. Before applying iron-based magnetic biochar for soil amendment, the material can be repeatedly cycled in biogas slurry treatment to recover nutrients such as nitrogen and phosphorus due to its recyclability. The adsorbed ammonia nitrogen and phosphorus can serve as potential nutrients for plants, thereby enhancing soil fertility and carbon sequestration capacity [60,61].

In summary, this study presents a unique perspective on the carbon cycle by enhancing carbon sequestration during the conversion of biomass into biochar, which is then applied to soil environments. This process not only aids in the sustainable management of waste but also holds potential for long-term carbon sequestration.

## 4. Materials and Methods

### 4.1. Pre-Treatment and Modification Methods for Mushroom Substrate

The spent mushroom substrate (SMS) samples were collected from the edible mushroom research base of Shanxi Agricultural University in China. Initially, the samples were air-dried and ground to pass through a 20-mesh sieve (<1 mm). A mixed solution of 0.5 mol/L HCl and 0.5 mol/L HF was used to rinse the biomass, thereby removing its inherent mineral content [62]. After the acid treatment, the biomass was washed with deionized water until the pH was adjusted to neutral. Subsequently, the treated material was dried in a vacuum oven at 105 °C to a constant weight, yielding demineralized spent mushroom substrate. Next, the demineralized samples were impregnated with a solution of ferric chloride (FeCl_3_). Specifically, 4.821 g of FeCl_3_ 6H_2_O was dissolved in 50 mL of deionized water to form a solution. After adding 10 g demineralized samples, the mixture was mechanically stirred until the samples were completely immersed in the solution. The mixture then remained at room temperature for 48 h. After impregnation, the remaining modified biochar was dried to a constant weight in a vacuum oven at 105 °C. For ease of distinction, the demineralized spent mushroom substrate with added mineral Fe was named DSMS-Fe, while the one without added mineral Fe was named DSMS.

### 4.2. Biochar Production and Characterization

#### 4.2.1. Biochar Preparation

Pyrolysis was conducted using a small microwave pyrolysis furnace (CY-OY1100C-S; Hunan Changyi Microwave Technology Co., Ltd., Changsha, China) with a heating power of 700W. The pyrolysis process was carried out under a nitrogen atmosphere, with a nitrogen flow rate of 200 mL/min. Samples were maintained at final temperatures of 300 °C, 400 °C, 500 °C, and 600 °C for one hour each. For simplicity, biochar samples derived from demineralized spent mushroom substrate were named DSMS_300_, DSMS_400_, DSMS_500_, and DSMS_600_, while those derived from demineralized spent mushroom substrate with Fe addition were named DSMS-Fe_300_, DSMS-Fe_400_, DSMS-Fe_500_, and DSMS-Fe_600_.

#### 4.2.2. Elemental and Ash Analysis

The contents of C, H, N, and S in the biochar were analyzed using an elemental analyzer (Vario EL cube, Elementar, Germany). The ash content of biomass and biochar was measured according to the GB/T6438-92 standard method. The content of O element is determined through the method of interpolation. The equation is as follows:

(1) $$O\;\left(\%\right)=100-C-H-N-S-Ash.$$

#### 4.2.3. Structure Characterization

The crystal composition of biochar surfaces was analyzed using an X-ray diffractometer (XRD, Ultima IV, Rigaku, Nanjing, China) within the range of 10–80°2θ with a step size of 2°·min^−1^. The crystallographic analysis of the biochar surfaces was conducted using MDI Jade 6.0 software. FTIR analysis of the biochar was performed using an FTIR spectrometer (FTIR-1500, Josvok, Tianjin, China), with all spectra recorded in the 400–4000 cm^−1^ region at a resolution of 4 cm^−1^. XPS (K-Alpha, Thermo Fisher, Shanghai, China) was employed to detect and semi-quantitatively analyze the C-containing functional groups on the biochar surface. Raman spectroscopy (LabRAM HR800, Horiba Jobin-Yvon, Paris, France) was used to analyze the carbon microstructure of the biochar. The pyrolysis process of biomass was monitored using a TG-MS analyzer (STA449F3-QMS403C, Netzsch, Selb, Germany). The thermogravimetric analyzer recorded the mass change signals of the samples during pyrolysis, and a mass spectrometer was used to analyze the volatile small molecule products in real time.

### 4.3. Biochar Carbon Retention and Stability

The carbon retention rate during pyrolysis and the carbon stability in the pyrolysis products were two crucial indicators for assessing the carbon sequestration capacity of biochar [23]. The formula for calculating the carbon retention rate is given as follows:

(2) $$Carbon\;retention\;\left(\%\right)=\frac{C_{biochar}}{C_{biomass}}\;Y_{biochar},$$

where *C_biochar_* and *C_biomass_* represent the carbon content (%) of biochar and biomass, respectively, and *Y_biochar_* denotes the yield (%) of biochar.

The readily oxidizable organic carbon of biochar was determined using the 333 mmol/L KMnO_4_ oxidation–colorimetric method [63]. The chemical oxidation method could remove unstable carbon components from the biochar while retaining stable carbon components, thereby evaluating the carbon stability of biochar.

### 4.4. Statistical Analysis

Origin 2022 was used to test the data obtained in the experiments. The least significant difference (*p* < 0.05) test was applied to assess the differences among the means of three replications.

## 5. Conclusions

The addition of exogenous mineral Fe during pyrolysis can result in approximately 10–40% more carbon being retained in biochar, and it effectively reduces the content of easily oxidizable carbon at temperatures of 300 °C, 400 °C, and 600 °C. Considering both carbon retention and carbon stability, the addition of Fe significantly enhances the carbon sequestration capacity of biochar during pyrolysis. This enhanced effect is strongly dependent on the pyrolysis temperature, as both Fe and pyrolysis temperature jointly influence the carbon sequestration capacity of biochar. This study explored the behavior of carbon in biochar derived from spent mushroom substrates during pyrolysis, including the decomposition and carbonization of carbon, its retention and release, and the microstructure of carbon at different pyrolysis temperatures. This research found that after loading exogenous Fe onto biochar via an impregnation–pyrolysis method, the Fe is converted into oxides, which act as physical barriers (Fe_x_O_x_) and form complexes by bonding with chemical bonds, thereby preventing the release of more organic small molecules (C_2_-C_5_). This phenomenon becomes more pronounced at higher temperatures. In summary, within the pyrolysis temperature range of 300–600 °C, the optimal carbon sequestration capacity of this type of Fe-containing biochar is achieved at 600 °C, reaching 60.6%. This study highlights the importance of Fe and pyrolysis temperature in enhancing the carbon sequestration capacity of biochar.

## Figures and Tables

**Table 1:** Basic properties of *Pleurotus ostreatus* substrate biomass and biochar.

Samples	Yield	Ash	C	H	N	S	O	Fe	H/C	O/C
	%	Wt %								
SMS	/	4.25	44.79	6.64	0.50	0.231	43.59	0.020	1.78	0.730
DSMS	/	4.01	47.42	7.87	0.45	0.255	39.99	0.011	1.99	0.633
DSMS_300_	48.1	5.69	61.24	5.33	0.59	0.260	26.89	0.030	1.04	0.329
DSMS_400_	31.4	6.62	67.37	2.30	0.92	0.112	22.68	0.047	0.41	0.252
DSMS_500_	27.6	9.9	74.25	1.63	0.71	0.326	22.68	0.040	0.26	0.229
DSMS_600_	25.2	8.52	80.73	1.11	0.77	0.238	8.63	0.047	0.16	0.08
DSMS-Fe	/	9.59	40.81	5.33	0.41	0.206	43.65	3.784	1.57	0.802
DSMS-Fe_300_	57.0	13.64	58.14	3.21	0.59	0.311	24.11	6.100	0.66	0.311
DSMS-Fe_400_	45.8	17.6	59.61	2.73	0.58	0.306	19.17	7.830	0.55	0.241
DSMS-Fe_500_	41.4	17.11	68.66	1.78	0.54	0.227	11.69	9.492	0.31	0.128
DSMS-Fe_600_	38.8	16.99	75.82	0.30	0.55	0.380	5.96	7.958	0.05	0.059

**Table 2:** Raman structural parameters.

	I(D)/I(G)	I(G)/I(All)
DSMS_300_	1.44	0.41
DSMS_400_	1.74	0.37
DSMS_500_	1.73	0.37
DSMS_600_	1.42	0.41
DSMS-Fe_300_	1.26	0.45
DSMS-Fe_400_	1.44	0.41
DSMS-Fe_500_	1.64	0.38
DSMS-Fe_600_	1.80	0.36

**Table 3:** Percent of the peak area as determined by XPS.

	C-C/C=C/%	C-O/%	C=O/%	O-C=O/%
DSMS_300_	74.37	15.64	4.41	5.58
DSMS_400_	77.58	8.15	6.80	7.46
DSMS_500_	78.05	9.00	5.76	7.19
DSMS_600_	78.10	12.472	1.72	7.49
	Increase	Decrease	Decrease	Increase
DSMS-Fe_300_	82.44	7.66	3.74	6.15
DSMS-Fe_400_	76.65	11.72	3.52	8.10
DSMS-Fe_500_	79.74	9.23	8.21	2.82
DSMS-Fe_600_	79.47	6.16	3.85	10.52

## Data Availability

The datasets generated during and/or analyzed during the current study are available from the corresponding author on reasonable request.
